# Editorial: Responses and adaptations to novel exercise modalities

**DOI:** 10.3389/fphys.2026.1782245

**Published:** 2026-01-14

**Authors:** Kevin D. Ballard, Kyle L. Timmerman

**Affiliations:** Department of Kinesiology, Nutrition, and Health, Miami University, Oxford, OH, United States

**Keywords:** Bajiquan, high intensity (strenuous) exercise, hypoxic acclimation, interval training, neuromuscular activation, yoga

## Introduction

Overwhelming scientific evidence supports the beneficial health and fitness effects of conventional exercise modalities, such as moderate-to-vigorous intensity aerobic (e.g., walking, cycling) and resistance training. Unconventional/novel exercise modalities are emerging as lifestyle approaches to enhance accessibility and long-term adherence, reduce common barriers to physical activity (e.g., lack of time, social influence, fear of injury), and improve health, fitness, and performance in a variety of individuals. Our Research Topic solicited studies performed to determine the physiological responses and adaptations elicited by unconventional exercise modalities that may enhance training, increase performance, and improve health outcomes. Several published studies included in our Research Topic are summarized below, which are divided into two categories: 1) Tech-Enhanced and Experimental Modalities, and 2) Traditional and Mind–Body Modalities.

## Tech-enhanced and experimental modalities

Several studies published in our Research Topic examined the effects of different interval training regimens on physiological responses and adaptations. Compared to traditional aerobic and resistance exercise programs, high-intensity functional training (HIFT) is more time efficient and involves multi-joint, high-intensity exercises performed to improve health- and skill-related physical fitness. Smith and colleagues (Smith et al.) utilized a randomized, crossover study design in 12 young, trained adults to compare the physiological responses between two HIFT workouts matched for volume and exercises performed: continuous-based with varied time domain (rounds for time = RFT; ∼12 min completion time) and interval-based with set time domain (every minute on the minute = EMOM; ∼20 min completion time). The EMOM workout was designed to ensure that participants had at least 30 s rest after each exercise, whereas the RFT workout was performed “all-out”. Compared to EMOM, RFT resulted in greater acute physiological stress as evidenced by higher heart rate, oxygen consumption (during and post-exercise), rating of perceived exertion, and blood lactate responses. Increases in serum creatine kinase at 24 h post-exercise did not differ between the workouts, indicating a similar degree of exercise-induced muscle damage. Both workouts are considered vigorous-intensity exercise that may be used to induce physiological adaptations, translating to improved health and performance. Future studies are needed to evaluate differences in training adaptations with high-intensity workouts varying in rest interval duration.

Bodyweight interval training (BW-IT) is a leading fitness trend promoted to increase exercise adherence and improve cardiometabolic health. Bellissimo and colleagues (Bellissimo et al.) used a single-group design to demonstrate in 14 inactive adults with obesity that a YouTube-instructed BW-IT program (3 days/week for 6 weeks) improved aerobic capacity, isometric muscular strength, and waist circumference. Body composition and biomarkers of cardiometabolic health (i.e., insulin resistance, blood lipids, C-reactive protein) were unchanged, suggesting that longer interventions may be needed to positively affect these health-related outcomes. The relatively small volume of independently performed BW-IT (20 min/session, 60 min/week) that led to improvements in health- and fitness-related outcomes is notable as lack of time and access to exercise facilities/equipment are common barriers to exercise among individuals with obesity.

Exercise intensity was also examined in adolescents. Su et al. compared sprint interval training (SIT) with moderate-intensity continuous training (MICT) in 24 male adolescents. Both 6 weeks interventions resulted in similar improvements in aerobic capacity. However, distinct metabolic adaptations were observed between training groups. SIT was associated with reduced circulating polyunsaturated free fatty acids, consistent with increased skeletal muscle lipid utilization. In contrast, MICT induced broader changes across multiple lipid classes, including sphingolipids and phospholipids. These findings suggest that while traditional cardiorespiratory fitness outcomes may be similar, exercise intensity differentially influences underlying metabolic remodeling in male adolescents.

Brandt et al. examined the physiological demands of Hyrox^©^, a rapidly growing hybrid fitness competition combining running with functional resistance tasks. Using a simulated competition format in 11 recreational Hydrox^©^ athletes, the authors reported sustained elevations in heart rate, blood lactate, and progressive increases in perceived exertion across successive exercise stations and running segments. Performance was strongly associated with endurance capacity, as athletes with greater aerobic fitness completed the event more efficiently, whereas measures of maximal handgrip strength were not predictive of overall performance. The structure of Hyrox^©^ shows how conventional aerobic and resistance exercises can be reorganized into novel formats that elicit substantial physiological stress and may appeal to a broad range of recreationally trained individuals.

Two studies published in our Research Topic examined the effects of electrical stimulation on neuromuscular function. Neuromuscular electrical stimulation (NMES) has been shown to improve muscle function and motor control in athletic and rehabilitation settings. Further, superimposing NMES onto voluntary muscular contractions (NMES+) results in greater improvements in muscle function and performance. Borzuola and colleagues (Borzuola et al.) investigated the acute neuromuscular effects of NMES+ (delivered to the tibialis anterior), passive NMES, and voluntary isometric contractions alone (ISO) in 17 young healthy adults. Experimental conditions involved 20 intermittent isometric ankle dorsi flexions performed at 20% of their maximum voluntary contraction. Surface electromyography was used to record myoelectric activity during steady force-matching contractions performed immediately after following each experimental condition. Compared to baseline levels and passive NMES, NMES + ISO increased motor unit discharge rate and the proportion of common synaptic input to spinal motor neurons and decreased the coefficient of variation of force (indicating improved force steadiness). Future research is needed to investigate the effectiveness of long-term NMES-based interventions to enhance rehabilitation and performance.

Research on whole-body electromyostimulation (WB-EMS) indicates enhanced body composition and muscle function. However, excessive/intense WB-EMS use may induce muscle damage. Knowledge is limited regarding the individual muscle damage response to intense WB-EMS. Teschler and colleagues (Teschler et al.) showed in 12 healthy adults that a single 20 min high-intensity WB-EMS training session (6 exercises, 4 sets x 5 reps) increased blood myoglobin and creatine kinase (CK) levels, peaking at 48 and 72 h, respectively. Individual peak myoglobin and CK responses to high-intensity WB-EMS were moderately predicted by post-WB-EMS blood lactate responses. Future research is warranted to determine factors that may explain differences in muscle damage temporal patterns following high-intensity WB-EMS, perhaps leading to individualized training and recovery sessions.

Finally, one study published in our Research Topic investigated adaptations to the innovative method of hypoxic training. Evidence is conflicting regarding enhancement of exercise training adaptations using innovative training methods, including the use of climate-controlled chambers to artificially induce hypoxia. Studies assessing the effectiveness of intermittent hypoxic training (IHT) (i.e., training in controlled hypoxic conditions while living in normoxic conditions) on exercise training adaptations in combat sports are lacking. Ambrozy and colleagues (Ambrozy et al.) utilized a randomized controlled trial in 20 elite-level boxers to demonstrate that 6 weeks of IHT (4 training sessions/wk at 230 m above sea level +4 training sessions/wk at a simulated altitude of 4,000 m) improved muscular endurance, anaerobic capacity, technical efficiency (i.e., number of punches delivered in 20 s), and post-exercise heart rate recovery compared to boxers who trained in normoxic conditions (8 training sessions/wk at 230 m above sea level). Training performed by both groups included aerobic, resistance, and technical and tactical boxing training (4 days/wk, 2 sessions/d (morning and afternoon), 480 min/wk). Future studies are needed to determine the potential performance benefits of innovative training methods for combat sports athletes.

## Traditional and mind–body modalities

Several studies in this Research Topic examined traditional, culturally-rooted, and mind-body exercise modalities that have gained renewed interest as accessible alternatives to conventional aerobic and resistance training. Collectively, these studies highlight how movement forms grounded in tradition or integrated mind-body practice can elicit meaningful physiological adaptations while also addressing enjoyment, adherence, and clinical applicability.

Wang and colleagues (Wang et al.) evaluated an 8-week Bajiquan training program performed 5–6 days/wk in 30 young adults. Compared with a control group engaging in general fitness activities of similar frequency and duration, Bajiquan training resulted in greater improvements in cardiovascular endurance, body composition, explosive power, and core strength. Participants also reported higher perceived social and personal benefits, emphasizing the potential for martial arts-based training to enhance both physical fitness and psychosocial engagement.

Chen et al. examined the acute cardiopulmonary responses to Baduanjin, a traditional Chinese mind-body exercise, in 30 patients with chronic heart failure, compared to steady-state cycle ergometry exercise performed at a matched oxygen consumption. Despite similar metabolic intensity between the two modalities, Baduanjin elicited a distinct physiologic response characterized by lower respiratory rate and minute ventilation, alongside differences in hemodynamic responses, including lower cardiac output and stroke volume with greater reliance on peripheral oxygen extraction. Additionally, the intermittent movement associated with Baduanjin elicited a non-steady state oxygen consumption profile, reflecting the dynamic muscular demands inherent to the practice. These findings suggest that Baduanjin may be a feasible alternative to light-to-moderate-intensity exercise for clinical populations, distributing physiological load across the respiratory, cardiovascular, and peripheral systems.

Mind-body exercise was further explored by Campbell and colleagues (Campbell et al.), who compared acute metabolic responses to a single session of vinyasa yoga versus a single session of matched-intensity cycling exercise in 12 young adults. While cycling elicited larger changes in urinary metabolites associated with glycolysis and oxidative metabolism, vinyasa yoga produced more modest but distinct metabolic responses, likely reflecting its distributed muscular workload and combined strength, balance, and flexibility demands. These findings suggest that although mind-body modalities may impose lower localized metabolic stress, they engage alternative physiological pathways that may complement traditional aerobic exercise.

Collectively, these studies demonstrate that mind-body exercise modalities can elicit measurable physiological and functional adaptations across diverse populations. Martial arts-based training and meditative movement practices demonstrated improvements in physical fitness, metabolic responses, and functional capacity, while also offering potential psychosocial and adherence-related benefits. Although the magnitude and nature of these variations varied by modality, intensity, and outcome assessed, the findings support the inclusion of culturally-rooted and integrative movement practices within evidence-based exercise programming and prescription. Further research is warranted to clarify underlying mechanisms, long-term adaptations, and optimal integration with conventional training approaches.

## Conclusion

Our Research Topic solicited studies assessing the responses and adaptations to unconventional exercise modalities, including those investigating tech-enhanced, experimental, and mind-body modalities. Further understanding of the responses and adaptations elicited by unconventional modalities, including those that merge emerging technology with conventional exercise modalities, may be applied to optimize training protocols, enhance performance, and improve human health. The diverse effects of unconventional modalities on physical and psychosocial health outcomes has broad public health implications and merit further exploration to develop individualized training protocols that maximize accessibility and improve overall health. Future mechanistic and longer-term studies are warranted to further elucidate differences between conventional and emerging, unconventional exercise modalities. We sincerely thank all authors and reviewers for their contribution to this Research Topic.

